# The complete chloroplast genome of *Clerodendrum lindleyi* Decne. ex Planch. (Tubiflorae: Verbenaceae) from nanjing, China

**DOI:** 10.1080/23802359.2021.1981785

**Published:** 2021-09-30

**Authors:** Jingxin Chen, Jiayu Chen, Long Wang, Yucheng Zhao, Minjian Qin

**Affiliations:** a School of Traditional Chinese Medicine, China Pharmaceutical University, Nanjing, China; b Department of Social Sciences, China Medical University, Shenyang, China

**Keywords:** Chloroplast genome, *Clerodendrum lindleyi*, phylogenetic analysis

## Abstract

*Clerodendrum lindleyi* Decne. ex Planch. is a Chinese medicinal plant in the Lingnan region of China. In this study, the complete chloroplast genome sequence of *C. lindleyi* was assembled and characterized from high-throughput sequencing data. The chloroplast genome is 151,678 bp in length, consisting of a large single-copy (LSC) and a small single-copy (SSC) regions of 83,043 bp and 17,311 bp, respectively, which are separated by a pair of 25,662 bp inverted repeat (IR) regions. The overall GC content of the genome is 38.18%. The genome contains 133 genes, including 88 protein-coding, 37 tRNA, and 8 rRNA genes. A phylogenetic tree reconstructed by using 16 chloroplast genomes reveals that *C. lindleyi* is most closely related to *C. trichotomum* which together forms a group that is a sister to genus *Caryopteris.* The work reported here is the first complete chloroplast genome of *C. lindleyi* which will provide useful information to the evolutionary studies on the genus of *Clerodendrum*.

*Clerodendrum lindleyi* Decne. ex Planch. is a kind of shrub with a special smell that can grow to 1–3 m. It is widely distributed in South China. Their dry roots and stems are used in Traditional Chinese Medicine (TCM) which have for dehumidification and detumescence in the Lingnan region of China (Chen et al. 2015). As a traditional Chinese medicine, it is widely used for treating bronchitis, hypertension, and rheumatism. Min et al isolated and characterized 13 bioactive compounds from *C. lindleyi*, including betulinic acid, monoacetylartinoside, clerodenoside A (Min et al. 2012). Until now, such studies have focused on the medicinal and insecticidal properties of these secondary compounds. However, the phylogenetic relationships of *C. lindleyi* remain unexplored and there is no genomic resources existing for research in this genus. In this study, the complete chloroplast genome of *C. lindleyi* was determined by using high throughput sequencing technology, which will provide valuable bioinformatic data for getter understanding the sytematics of *Clerodendrum* genus and forms the foundation for future genetic research.

The fresh leaves of *C.lindleyi* were sampled from Nanjing city, Jiangsu Province, China (31°54′10.37″N, 118°55′5.12″E). Specimens were stored in the Medical Botanical Garden of China Pharmaceutical University (accession number: Qin-JCCML-07). Total genomic DNA was extracted with a modified CTAB protocol according to Doyle and Doyle (1987). The whole genome sequencing was conducted by Hefei Biodata Biotechnologies Inc. (Hefei, China) on the Illumina Hiseq platform. The filtered sequences were assembled by using the program SPAdes assembler 3.10.0 using default settings (Bankevich et al. 2012). The annotation was performed with DOGMA (Wyman et al. 2004) and BLAST searches.

The chloroplast genome of *C.lindleyi* is 151,678 bp in length (GenBank accession no. NC_056199) and contains inverted repeat (IR) regions of 25,662 bp, respectively, separated by large single-copy (LSC) and small single-copy (SSC) regions of 83,043 bp and 17,311 bp. The overall GC content of the *C.lindleyi* complete chloroplast genome is 38.18%, and the corresponding values in LSC, SSC and IR regions are 36.3%, 32.1%, and 43.3%, respectively. The complete chloroplast genome was predicted to contain 133 genes, including 88 protein-coding, 37 tRNA, and 8 rRNA genes. Eight protein-coding, six tRNA, and four rRNA genes were duplicated in the IR regions. Nineteen genes contained two exons and four genes (*clp*P, *ycf*3, and two *rps*12) contained thee exons.

To investigate its taxonomic status, alignment was performed on the 16 complete chloroplast genome sequences using MAFFT v7.307 (Katoh and Standley 2013), and a Maximum Likelihood (ML) tree was constructed with 1000 bootstrap replicates in FastTree v2.1.10 (Price et al. 2010) with the GTR + Gamma model. *Scrophularia ningpoensis* and *S. henryi* were used as outgroups to construct the phylogenetic tree. As expected, *C. lindleyi* is fully resolved in a clade sister to *D. trichotomum* and the *Clerodendrum* forms a group that is a sister of the genus *Caryopteris* (Figure 1). The complete chloroplast genome sequence of *C. lindleyi* will provide a useful resource for the conservation genetics of this species as well as for the phylogenetic studies of Verbenaceae.

**Figure 1.**
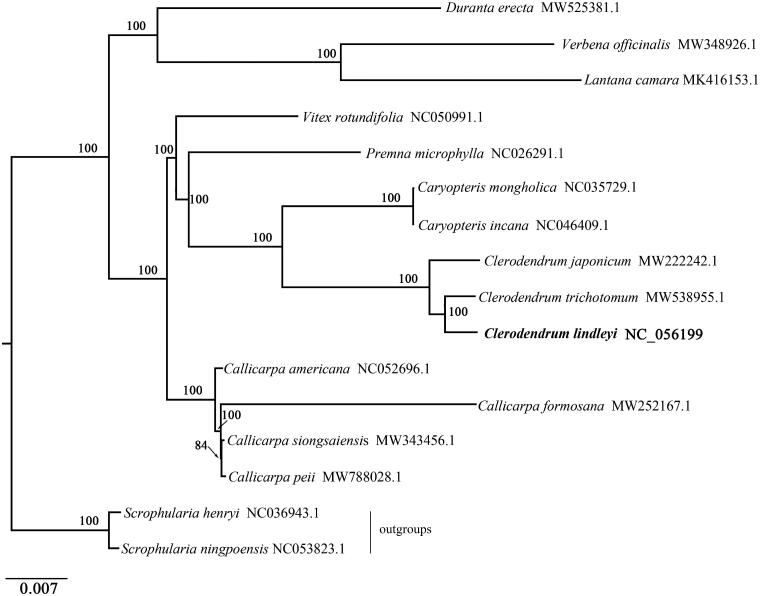
Phylogenetic tree inferred by Maximum Likelihood (ML) method based on the complete chloroplast genome of 16 representative species. Bootstrap support (*N* = 1000) values are indicated at each node and GenBank accession numbers follow each species name..

## Data Availability

The genome sequence data that support the findings of this study are openly available in GenBank of NCBI at https://www.ncbi.nlm.nih.gov/ under the accession no. NC_056199. The associated BioProject, SRA, and Bio-sample numbers are PRJNA685332, SRS7884001 and SAMN17082918 respectively.
